# Pharmacological activities and molecular mechanisms of *Pulsatilla* saponins

**DOI:** 10.1186/s13020-022-00613-8

**Published:** 2022-05-23

**Authors:** Jinmiao Zhong, Lihua Tan, Meiwan Chen, Chengwei He

**Affiliations:** 1 State Key Laboratory of Quality Research in Chinese Medicine, Institute of Chinese Medical Sciences, University of Macau, Taipa, 999078 Macao SAR China; 2 Department of Pharmaceutical Science, Faculty of Health Sciences, University of Macau, Taipa, 999078 Macao SAR China; 3 Guangdong-Hong Kong-Macau Joint Lab on Chinese Medicine and Immune Disease Research, University of Macau, Taipa, 999078 Macao SAR China

**Keywords:** *Pulsatilla chinensis*, Saponins, Anticancer, Anti-inflammatory, Organ protection

## Abstract

Saponins are found in a variety of higher plants and display a wide range of pharmacological activities, including expectorant, anti-inflammatory, vasoprotective and antimicrobial properties. *Pulsatilla chinensis* (*P. chinensis,* Bai Tou Weng, 白頭翁) has been used medically in China for thousands of years for the treatment of diseases caused by bacteria, and it is rich in triterpenoid saponins. In recent decades, anemoside B4 (Pulchinenoside C) is well studied since it has been used as a quality control marker for *P. chinensis*. At the same time, more and more other active compounds were found in the genus of *Pulsatilla*. In this review, we summarize the pharmacological activities of *Pulsatilla* saponins (PS) and discuss the cellular or molecular mechanisms that mediate their multiple activities, such as inducing cancer cell apoptosis, inhibiting tumor angiogenesis, and protecting organs via anti-inflammatory and antioxidant measures. We aim to provide comprehensive analysis and summary of research progress and future prospects in this field to facilitate further study and drug discovery of PS.

## Introduction

For decades, natural compounds isolated from herbal and animal medicines have been an important source for potential new drugs [1, 2], such as saponins [3], flavonoids [4] and alkaloids [5]. Among them, natural saponins are a kind of compounds with great research value. The name “saponin” is derived from the Latin word *sapo*, meaning soap-like foam-generating ability, which is because saponins are surfactants. In the chemical structure of saponins, aglycones have different degrees of lipophilic properties and sugar chains have strong hydrophilic properties, which render persistent soap-like bubbles being produced after the aqueous solution is shaken. Saponins are a class of secondary metabolites that found in plants and marine animals, which can be divided into two main groups [6, 7]: triterpenoid saponins and steroid saponins. Triterpenoid saponins are a class of terpenoids with 30 carbon atoms assembled from six five-carbon isoprene units, which are widely distributed in dicotyledons, including four major subclasses: pentacyclic oleanane, ursane, lupane, and tetracyclic dammarane. Steroid saponins are mostly derived from monocotyledons, also including four major subclasses: tetracyclic cholestane, hexacyclic spirostane, pentacyclic furostane, and lactone-bearing cardenolide. Many saponins shown great potential anti-cancer activity, and some of them have been found against neurodegenerative diseases, cardiovascular disease and kidney disease. It is worth noting that triterpene saponins exhibit selective action on tumor and normal cells, possessing a high efficiency in inhibition of cancer cell growth [8]. Previous studies suggested that saponins could  serve as promising leading compounds for the development of natural product-derived drugs.

The genus of *Pulsatilla* contains about 40 species of perennial herbaceous plants native to meadows and prairies of North America, Europe, and Asia. *Pulsatilla* decoction has been used medically in China for the treatment of diseases caused by bacteria for thousands of years, and *Pulsatilla chinensis* (*P. chinensis*, Bai Tou Weng, 白頭翁) is a chief herbal source of it. All the previous studies mainly focus on *P. chinensis* and *Pulsatilla koreana*. With the extensive research to *Pulsatilla*, an increasing number of biological activities were discovered. However, the detailed mechanisms for these activities of *Pulsatilla* extract remain largely unknown. Saponins are thought to be the major active components of *Pulsatilla* [9–11], such as pulchinenoside A (PSA or anemoside A3, AA3), pulchinenoside C (PSC or anemoside B4, AB4), *pulsatilla* saponin D (PSD or SB365) and pulsatilloside E (PSE). Besides, PSA and 23-hydroxybetulinic acid are two possible metabolites of PSC, and among triterpenoid saponins, PSC is the most abundant component in *Pulsatilla* and has been used as a quality control marker for *P. chinensis*. PSC is believed to be the principal active ingredient of *P. chinensis*. Afterwards, PSD has been identified and well-known because of its remarkable anticancer activity against hepatocellular carcinoma, HeLa cells [12], and colon cancer cells [13].

**Fig. 1:**
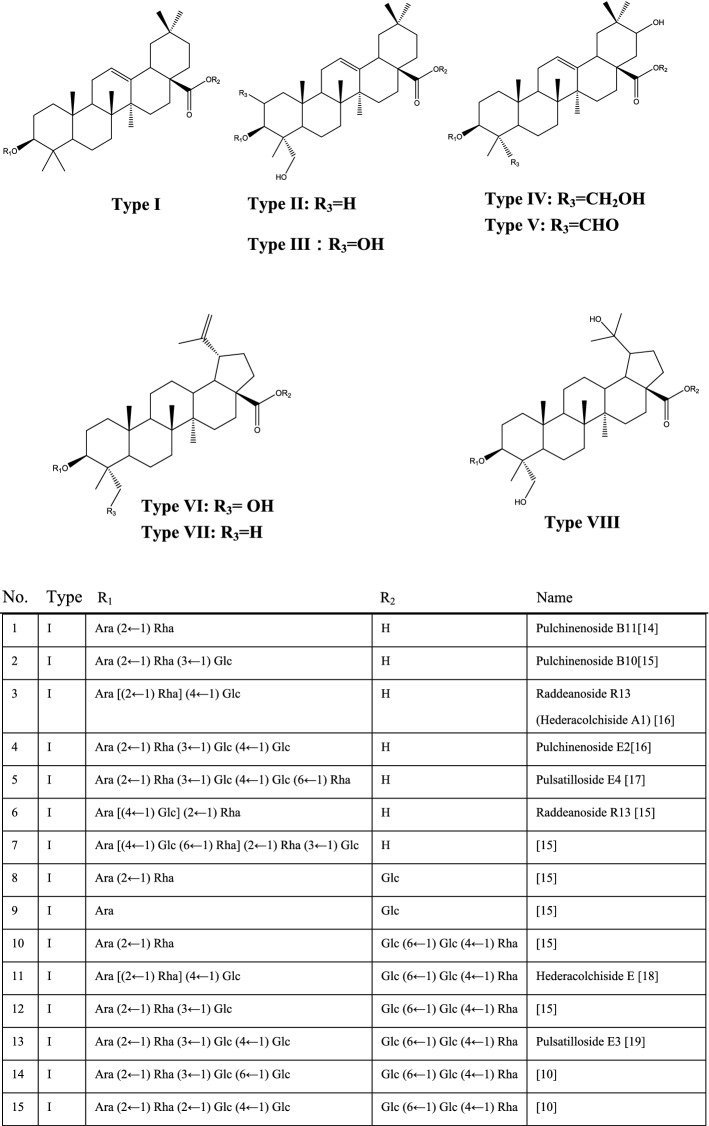

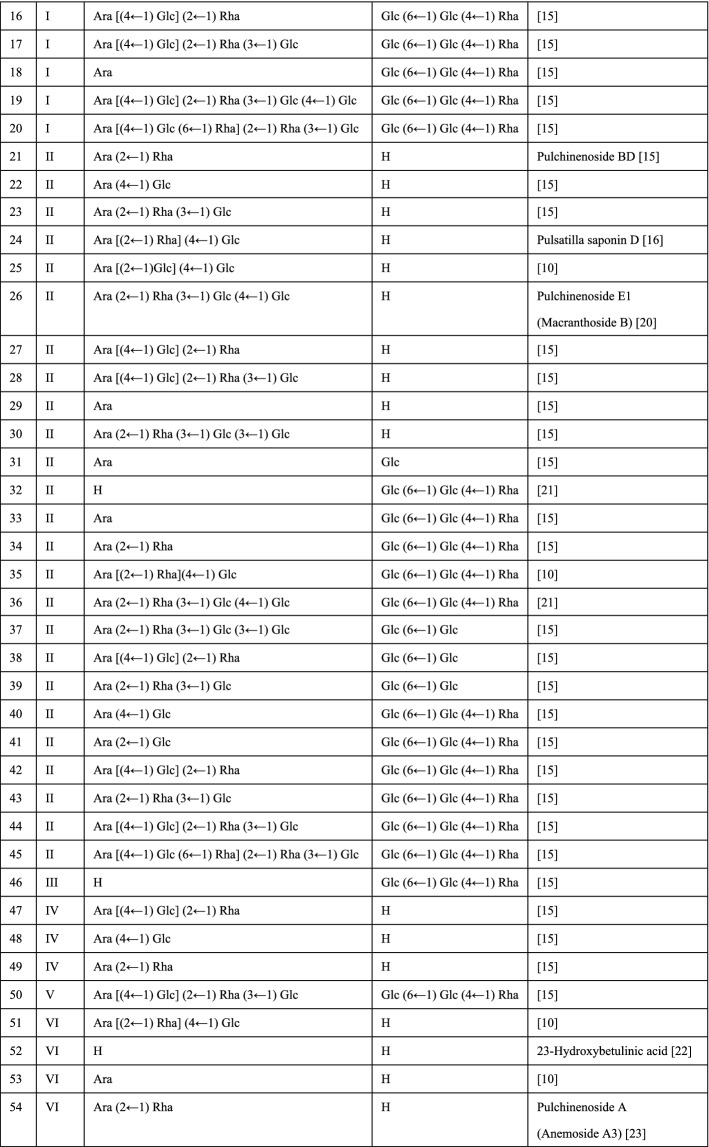

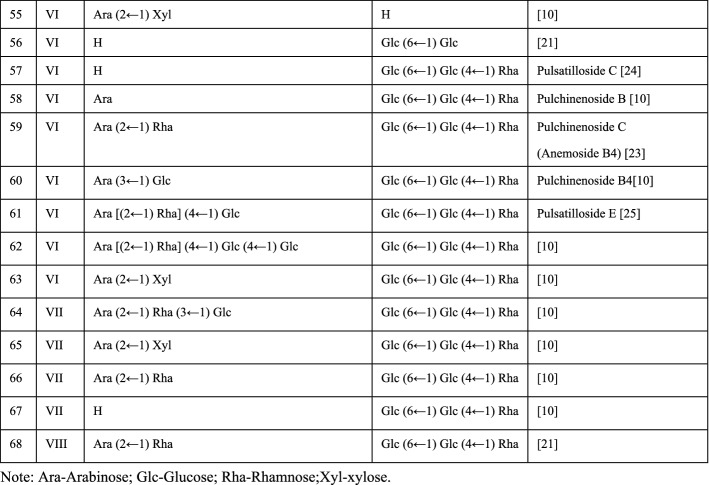
Saponins extracted from the genus of *Pulsatilla*

*Pulsatilla* is rich in saponins as listed in Fig. 1. The regulatory effects of triterpenoid saponins from *Pulsatilla* on apoptosis, autophagy, cell proliferation, and immunity have been extensively studied. The PS is basically divided into eight types (Fig. 1) base on the skeletons, and the carbohydrate chains are connected to C-3 (Fig. 1R1) and C-28 (Fig. 1R2). C-28 is generally connected to H, Glc and Glc (6 ← 1) Glc (4 ← 1) Rha, and the monosaccharide molecules (Ara, Glu, Rha and Xyl) at C-3 are connected in various ways (Fig. 1). The therapeutic potential and underlying mechanisms of many of these saponins remain to be profoundly investigated. In this review, we aim to summarize the current progress in the pharmacological activities and mechanisms of *Pulsatilla* extract and particularly its saponins.

## The anticancer activity

Cancers have been the top threat to human health. Epidemiological data clearly show an increase of cancer prevalence all over the world. It is imperative to develop new drugs for effective cancer treatment. In recent years, there are increasing numbers of research focusing on the anticancer function of Chinese herbal medicine due to their lower toxicity and multiple targets compared to chemotherapeutic drugs [21]. *P. chinensis* was firstly found to have anticancer activity in 1996 [22]. Over the past years, the saponins from the genus of *Pulsatilla* have been proved to to be the active ingredients which could act on diverse cancer cell types through various mechanisms.

### Regulating apoptosis

Cell apoptosis can be initiated by two mechanisms, including the death receptor-mediated extrinsic and mitochondria-mediated intrinsic apoptotic pathways. Although the two pathways are different, both ultimately lead to caspase-3 activation. The related genes and signaling pathways regulating apoptosis of cancer cells involve Bcl-2, caspases, p53 and PI3K/Akt/mTOR signaling, etc. [26]. Bcl-2 and caspase family members are the downstream targets of Erk pathway during regulating apoptosis. Bax acts as a pro-apoptotic protein that promotes the release of cytochrome Cinto mitochondria and activates caspase-3 that is the major executor of apoptosis. In contrast, Bcl-2 protein antagonizes Bax and inhibits the initiation of apoptosis. For p53, it plays an important role in triggering apoptosis and p53-dependent apoptosis is induced by DNA damage, hypoxia, withdrawal of growth factors [26]. PI3K/Akt/mTOR pathway is an important intracellular signaling that regulates cell proliferation, survival, migration and so on. Many studies indicated the PI3K/Akt/mTOR pathway regulates cancer cell apoptosis [27].

**Fig. 2:**
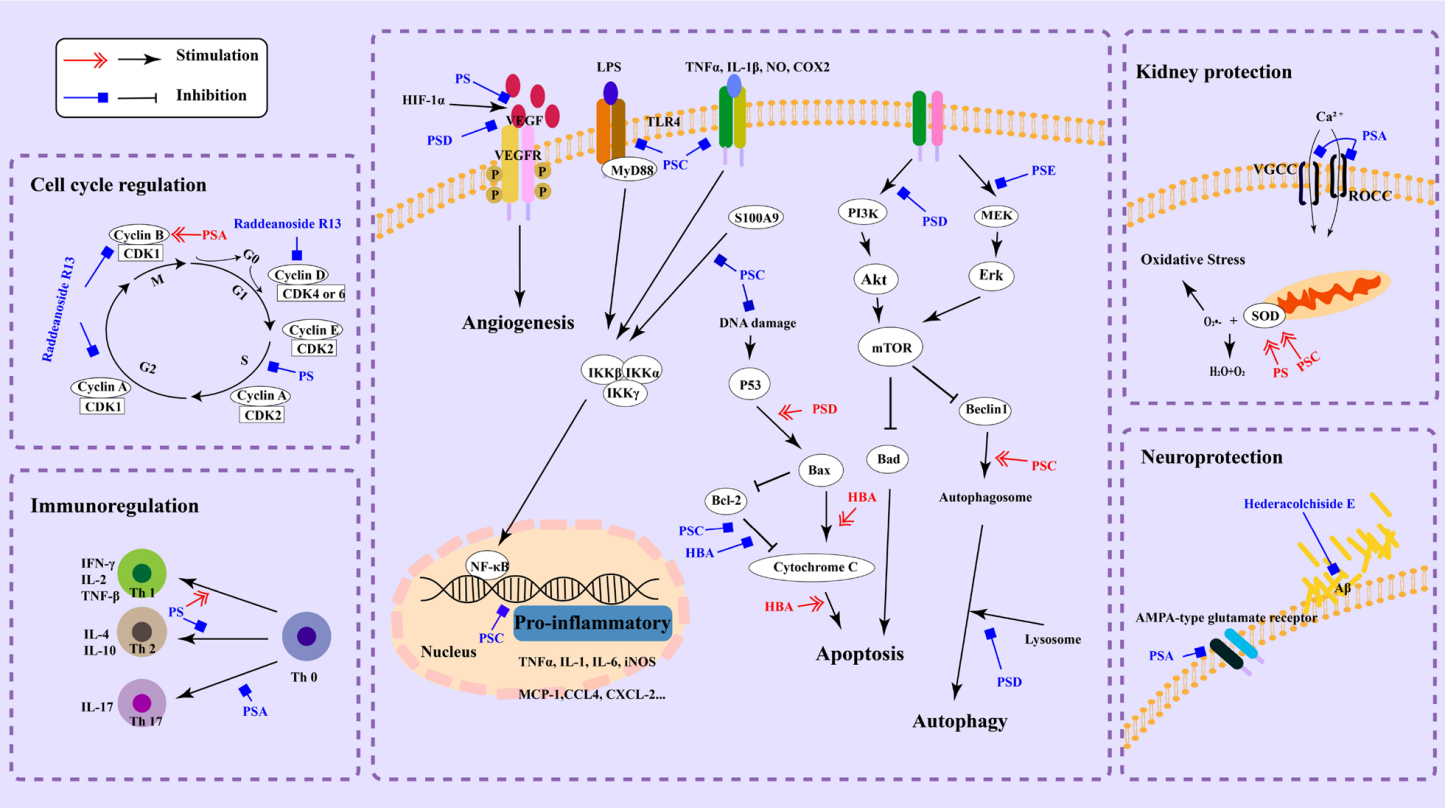
The molecular mechanisms for the pharmacological activities of *Pulsatilla* Saponins

Many studies demonstrated that PS could induce apoptosis in cancer cells through regulating multiple signaling pathways (Fig. 2). Xue et al. reported that AB4 inhibited the growth of hepatocellular carcinoma (HCC) SMMC7721 cells and strongly induced apoptosis through manipulating the Bcl-2-caspase-3 pathway, the increased protein level of Bax and cleaved caspase-3 and the decreased level of Bcl-2 were simultaneously observed [28]. In addition, according to the collected data, PSA is more likely to target Bcl-2/Bax-caspase-3 signaling pathway than any other pathways. For example, PSA has been found to significantly inhibit the growth of HCC SMCC-7721 cells, pancreatic BXPC3 and SW1990 cancer cells [29], and similar inhibitory activities were also verified when PSA was tested in mouse xenograft tumor models using HCC Bel-7402 and pancreatic SW1990 cancer cells. Besides, PSA may exert its antitumor effect by inducing DNA damage and apoptosis of cancer cells simultaneously, since p53 and cyclin B protein levels were higher, whereas Bcl-2 protein levels were lower in PSA-treated cancer cells. The DNA damage may relate to caspase-3-cleaved poly (ADP-ribose) polymerase (PARP) that eventually leads to DNA fragmentation [30]. The aglycone 23-hydroxybetulinic acid (HBA) significantly disrupts mitochondrial membrane potential, selectively decreases the levels of Bcl-2 and surviving, and upregulates Bax, cytochrome C and cleaved caspase-3 and 9 [31]. Another study reveals the benefits of combination drug therapy, when treated with PSD, Raddeanoside R13 and PSA at the same time, more apoptosis in NCI-H460 cells was induced comparing with single drug administration [32]. The activation of Bcl-2/Bax-caspase-3 signaling pathway caused by PS has been observed in HCC, pancreatic cancer, myelogenous leukemia and lung cancer.

The PI3K/Akt/mTOR is the classical signaling pathway in inducing apoptosis. SB365 (PSD) can significantly inhibit tumor growth in an HCC xenograft model and induce apoptosis by effectively suppressing the phosphorylation of PI3K downstream factors, such as Akt, mTOR and p70S6K both in vitro and in vivo [33]. In addition, SB365 also suppresses the proliferation of human colon cancer cells and induces apoptosis by inhibiting the AKT/mTOR signaling pathway, leading to the suppression of tumor growth and angiogenesis [13]. To further identify the effect of SB365 on c-Met/Akt/mTOR pathway, researchers found SB365 treatment can reduce the levels of p-c-Met, p-Akt, p-mTOR and p-p70S6K in MKN-45 gastric cancer cells [34]. The activation of mTOR results in the phosphorylation of effectors such as p70S6K, leading to mTOR-dependent gene transcription, which would regulate cell proliferation and protein synthesis. Besides, the phosphorylation of mTOR and p70S6K was also inhibited by PSD in cervical cancer Hela cell [12].

### Regulating autophagy

Autophagy is an intracellular recycling pathway with implications for intracellular homeostasis and cell survival, and is regulated by autophagy-related proteins (ATGs) and microtubule-associated protein light chain 3 (LC3), Beclin-1, P62 [35] and a group of highly regulated signaling pathways, such as mTOR signaling pathway, Class I PI3K/PKB and Class III PI3K signaling pathway. The roles of autophagy during tumorigenesis and cancer treatment are complex. The turnover of LC3-II from cytosolic LC3-I is a marker of autophagosome formation during the process of autophagy. In recent years, studies demonstrated that saponins, including PS, can inhibit tumor growth by regulating autophagy.

For instance, PSD promoted the formation of autophagosomes, however, it inhibited the autophagy–lysosomal degradation and consequently inhibited autophagic flux in HeLa cells [12]. Besides, the decreasing expression of ERK1/2 caused by PSE was also found in NSCLC [36], ERK-dependent autophagic activity is associated with classical markers of autophagy [37], such as the conversion of LC3-I to LC3-II, and ERK-mediated phosphorylation of p53 on serine 392 was also involved in TNFα-induced autophagy (Fig. 2). For breast cancer cell lines (MCF-7 and MDA-MB-231), PSD has also been proved to inhibit autophagic flux by interrupting autophagic-lysosomal function and promoting p62-mediated ubiquitinated protein aggregation [38]. Collectively, the previous studies indicated that PSD could inhibit protective autophagy and thereby enhance the sensitivity of cancer cells to chemotherapeutic drugs.

However, Raddeanoside R13 could induce autophagy and induce cell cycle arrest, apoptosis, and reversion of epithelial-mesenchymal transition (EMT) in breast cancer cells (ZR75-1 and MCF-7) [39]. In addition, AB4 treatment simultaneously induced apoptosis and autophagy through PI3K/Akt/mTOR signaling in hepatocellular carcinoma (HCC) cells [28]. PSD and PSE are more likely act as an autophagy-inhibitor while AB4 and Raddeanoside R13 are autophagy inducers according to the previous studies, but they all show anticancer activity. In fact, autophagy is believed to suppress tumorigenesis but promote tumor growth by helping tumor cells survive and increasing stress tolerance and drug resistance [40].

### Inhibiting angiogenesis

Hypoxia-inducible factor α (HIF-1α) plays a central role in tumor progression and angiogenesis, mainly acting as a transcriptional modulator of angiogenic factors such as vascular endothelial growth factor (VEGF). Cell migration is critical for endothelia cell to form blood vessels in angiogenesis and is necessary for tumor growth and metastasis.

The expression of HIF-1α and VEGF can be increased under hypoxic conditions. A study reported that *P. koreana* extract (PKE) treatment at 50–300 μg/ml markedly inhibited the hypoxia-induced HIF-1α expression in a dose-dependent manner. The treatment of PKE inhibited hypoxia induced VEGF expression in human umbilical vein endothelial cells (HUVEC). Besides, PKE could inhibit the formation of vessel-like structures, and the PKE treatment remarkably inhibited wound healing in wound migration assay [41]. PKE could down-regulate the expression of HIF-1α and VEGF in tube formation assay, also inhibit the formation of vessel in vivo [42]. These results indicated that PKE could prevent tube formation and migration of endothelial cell, suggesting that PKE has a potent anti-angiogenic property. PSD, the main active compound in PKE, exhibited the similar potent anti-angiogenic activity like PKE. In addition to decreasing HIF-1α and VEGF [13], PSD could suppress the tube formation and migration of HUVEC, as well as in vivo neovascularization in a mouse matrigel plug assay [33] (Fig. 2).

### Regulating cell cycle

PSA exerted its anticancer effect by inducing DNA damage and causing G2 arrest and apoptosis in HCC SMCC-7721 cells, pancreatic BXPC3 and SW1990 cancer cells, with an increase of p53 and cyclin B levels [29]. Raddeanoside R13 inhibited breast cancer cell (ZR75-1 and MCF-7) proliferation via activating Gl/S checkpoint transitions and markedly decreased cell cycle regulators, including cyclin D1, cyclin A, and cyclin B1 [39]. The total pentacyclic triterpenoid saponins from *Pulsatilla* extraction can significantly inhibit the invasion and migration of SW480 cancer cells and block the cell cycle at S phase [43]. These studies suggested that the PS are not cell cycle specific agents (Fig. 2).

### Others

The total saponins from *Pulsatilla* extraction hindered TGF-βl-induced EMT, diminished migration and invasion of SW480 cells, and also promoted apoptosis with decreased expression of MMP-9, CYP2C9, CYP2C19, CYP3A, and N-cadherin [43]. PSA could induce the differentiation of U937 cells, K562 cells and HL-60 cells, modify the differentiation activity of the acute myelocytic leukemia cells probably via the MEK/Erk signaling pathway [44].

PSD exerted a cytotoxic effect on U87-MG and T98G glioblastoma multiforme cells not by inducing apoptosis, as was observed in other cancer cell lines, but by triggering caspase-independent cell death. Inhibition of autophagic flux and neutralization of the lysosomal pH occurred rapidly after application of PSD, followed by deterioration of mitochondrial membrane potential. A cathepsin B inhibitor and N-acetyl cysteine, an antioxidant, partially recovered cell death induced by PSD. PSD in combination with temozolomide exerted an additive cytotoxic effect in vitro and in vivo [45]. PSE has been shown to suppress viability, migration and invasion of NSCLC through negatively regulating Akt/fatty acid synthase (FASN) signaling pathway via the inhibition of flotillin-2 in lipid raft [36].

## The anti-inflammatory activity

Inflammation is necessary for the preservation of the integrity of the organism in the event of harmful damage [46]. Inflammatory diseases majorly altere the development of response of T cells, causing dysregulation of immune function which starts with the migration of leukocytes and primarily neutrophils [47]. During inflammatory response, the pro-inflammatory molecules such as tumor necrosis factor α (TNF-α), interleukin-1β (IL-1β), and IL-6 are largely produced by macrophages. The inflammatory response is transduced by a variety of signaling pathways. Among of them, nuclear factor kappa B (NF-κB) is one of the essential signaling molecules acting as a transcription factor for the expression of inducible nitric-oxide synthase (iNOS), TNF-α and IL-6 genes by transactivation. The anti-inflammatory activities of *Pulsatilla* saponins basically rely on the following mechanisms (Fig. 2).

The TNF-α/NF-κB signaling is the most important pathway in inflammatory response. To investigate the pharmacological mechanism, PSD and AB4 down-regulated TNF-α expression in rat intestinal microvascular endothelial cells [48]. AB4 is a quality control marker for *P. chinensis* with amount of over 4.6% (W/W) in the herb [3]. Study proved that 12.5–50 mg/kg AB4 could significantly suppress xylene-induced mice ear edema. Furthermore, it ameliorated LPS-induced kidney and lung damage through inhibiting NF-κB-mediated pro-inflammatory response in mice [49]. In addition, AB4 showed significant protective effect on acute kidney injury induced by cisplatin in mice. It mainly acted on NF-κB signaling pathway to reduce the levels of TNF-α, IL-1β, cyclooxygenase-2 (COX-2) and iNOS, thus exerting anti-inflammatory activity [50]. Further, Quantitative proteomic analyses discovered that 56 proteins were significantly altered by AB4 in 2,4,6-trinitrobenzene sulfonic acid induced colitis rat model, among all the proteins, S100A9 is one of the most significantly down-regulated proteins and associated with NF-κB and MAPK signaling pathways in the pathogenesis of ulcerative colitis. In addition, AB4 suppressed the expression of S100A9 downstream genes including TLR4 and NF-κB in colon tissue [51].

Early study found the saponins isolated from *P. koreana* showed significant activity of inhibiting NO production in LPS-stimulated RAW 264.7 cells [52]. Moreover, these oleanane-type triterpenoid saponins showed different degree of activities in inhibiting TNFα-stimulated NF-κB activation, the expression of iNOS and ICAM-1 mRNA and the activation of PPARs in a dose-dependent manner [53]. In addition, evidences shown that AA3 treatment significantly reduced the severity and inflammatory infiltration in the spinal cord of experimental autoimmune encephalomyelitis (EAE) mice by regulating prostaglandin E receptor 4 signaling [54]. Also, AA3 could inhibit the T cell response toward the encephalitogenic epitope of myelin oligodendrocyte glycoprotein (MOG) and significantly downregulate the expressions of certain Th1 and Th17 cytokines in activated T cells re-stimulated by MOG. Moreover, AA3 inhibited the activation of STAT4 and STAT3 which are the transcription factors pivotal for Th1 and Th17 lineage differentiation, respectively. Pharmacological analysis further indicated that AA3 reduced Th17 cell differentiation and expansion.

## The antioxidant activity

Oxidative stress is a result of an imbalance between ROS production and antioxidant defense mechanisms. Oxidative stress causes a series of cellular dysfunctions and leads to various pathological conditions, leading to oxidative modification of biological macromolecules, tissue injury, and accelerated cell death, which are the foundation of many diseases [55]. Antioxidants play a vital role in human body to reduce oxidative processes and harmful effects of ROS [56, 57].

The antioxidant activity of PS involves different mechanisms. Studies indicated that the treatment of *P. chinensis* extract can specifically increase superoxide and markedly increase mitochondria MnSOD activity in liver tissue at the same time, which may prevent possible infections and superoxide-mediated toxicity [58].

Seo et al. reported that SK-PC-B70M, an oleanolic-glycoside saponin-enriched fraction derived from the *P. koreana*, inhibited malondialdehyde (MDA) and 4-hydroxy-2-nonenal (HNE), the products of lipid peroxidation, in hippocampus of mice [59]. Further studies showed that SK-PC-B70M treatment also significantly decreased the levels of MDA and HNE in the spinal cord of amyotrophic lateral sclerosis (ALS) mouse model [60]. AB4 potently increased cisplatin-treated HEK 293 T cells viability and inhibited cells apoptosis, which may be due to the decreased ROS content and improved SOD activity [61].

## The immunomodulatory activity

PS could enhance the cellular specific immune response to ovalbumin (OVA) in mice and significantly increase the level of specific IgG antibodies in mice immunized with OVA. In addition, the level of cytokines IL-2 and interferon γ (IFN-γ) was also significantly increased in immunized spleen cells treated with PC [21]. The Th1 immune response is mediated by Th1 helper cells, which is characterized by production of the cytokines IL-2, TNF-β and IFN-γ. PS may mainly trigger the Th1 type immune response, which is frequently effective against intracellular pathogens and malignant cells. AB4 inhibited the production of IL-4 and IL-10 and increased the secretion of IFN-γ and IL-2 in pigs. IL-4 and IL-10 are important Th2 cytokines key to humoral responses and IFN-γ and IL-2 are Th1 cytokines that have an important role in cellular immune response. These data suggested that AB4 might effectively regulate immune responses via regulating the production of certain cytokines [62].

## The organ protective activity

AA3 relaxes rat renal arteries mainly by inhibiting Ca^2+^ influx through L-type voltage-gated Ca^2+^ channel (VGCC) and receptor-operated Ca^2+^ channel (ROCC) (Fig. 2), while endothelium-derived hyperpolarizing factor and K^+^ channel activation in vascular smooth muscle cells play a minor role. AA3 could be applied to the treatment of renovascular hypertension by reducing vascular tension [63]. As a first-line chemotherapy agent, cisplatin also has toxic side effects on the kidney, and AB4 showed detoxicating effect against cisplatin-caused body weight loss and kidney injury without reducing anticancer activity of cisplatin [61]. It could regulate MAPK signaling pathway and its downstream apoptotic factors to inhibit the inflammatory and apoptosis that induced by cisplatin [50]. In addition, AB4 could increase urinary adenine contents and promote its excretion, and upregulate the expression of podocin and nephrin, two major podocyte proteins, and reduce the fiber collagen in the renal interstitial [64], which suggesting that AB4 could protect the glomerular matrix from adenine injury.

Hederacolchiside E shows a strong neuroprotective effect against Aβ induced neurotoxicity. The neurotoxic events caused by Aβ include an increase in the levels of ROS and neuronal inflammation. The neuroprotective effect of Hederacolchiside E could be attributed to the anti-inflammatory and antioxidant activities [65, 66]. SK-PC-B70M improved impairments in memory consolidation and spatial memory which induced by systemic injection of scopolamine, a muscarinic cholinergic receptor antagonist. The rats treated with both scopolamine and SK-PC-B70M had significantly less search error compared with the rats with scopolamine only [67]. In further study, SK-PC-B70M could reduce plaque formation in the brain and up-regulate the expression of transthyretin, phospho-ERK, and phospho-CREB. In addition, SK-PC-B70M suppressed the neuronal toxicity induced by H_2_O_2_ in primary cortical culture and notably reduced the levels of products of lipid peroxidation in the hippocampus, such as MDA and HNE [59]. AA3 can specifically increase serine phosphorylation within GluAI, a subunit of AMPA (α-amino-3-hydroxyl-5-methyl-4-isoxazole-propionate)-type glutamate receptors, which is necessary for the trafficking of AMPA-containing GluAI to synapses (Fig. 2). Furthermore, AA3 administration activates several synaptic signaling molecules and increases protein expression of the brain-derived neurotrophic factor (BDNF) and monoamine neurotransmitters in the mouse hippocampus. AA3 also acts as a non-competitive *N*-methyl-d-aspartate (NMDA) receptor modulator with a neuroprotective capacity against ischemic brain injury and overexcitation in rats [68]. These results suggest that some PS can be cognitive enhancers capable of alleviating memory dysfunctions associated with aging and neurodegenerative diseases.

## The anti-microorganism activity

In an ancient Chinese medicine book *Treatise on Febrile and Miscellaneous Diseases* (Shang Han Za Bing Lun, 傷寒雜病論), it has been recorded that *P. chinensis* has anti-dysenteric activity, and it is currently used for the treatment of digestive infections including enteritis, bacillary dysentery, and intestinal amoebiasis in clinical application [3]. In modern research, studies found that the root of *P. chinensis* showed antibacterial effect on *Staphylococcus aureus*, *Staphylococcus albus*, *Pseudomonas aeruginosa*, *Bacillus anthracis*, *Bacillus typhoid*, *Streptococcus A* and *Streptococcus B* [70]. The total saponins of *P. chinensis* also show bacteriostatic effects on *Staphylococcus aureus*, *Pseudomonas aeruginosa*, *Paratyphoid bacillus* and *Escherichia coli* [70], suggesting that the saponins might be the main components for the antibacterial activity. PKE showed growth inhibition against Gram positive (*S. aureus, S. epidemidis, B. subtilis, M. luteus, L. plantarum, and L.mesenteroides*) and Gram negative (*E. coli, S. typhimurium, P. aeruginosa and V. vulnificus*) bacteria and yeasts (*S. cerevisie and C. albicans*) [71]. However, although the antibacterial activity of PS has been continuously discovered and verified, there are still few studies on the antibacterial mechanisms. In recent year, studies found that the powder of *P. chinensis* root can be effective in the treatment of *E.coli* O101-induced diarrhea in mice. As the study reported, it can improve the weight loss caused by diarrhea, increase spleen and thymus indices, and reduce the diarrhea index. In addition, it also reduced the number of white blood cells, regulated the level of cytokines, and regulated the intestinal microbial flora [72].

In addition, components from *Euphorbium resinifera* and *P. pratensis *show antiviral activity against respiratory syncytial virus [73]. AB4 could suppress the influenza virus FM1 strain-induced expression of toll-like receptor 4 (TLR4), myeloid differential protein-88 (MyD88) and myeloid differentiation protein-2 (MD-2). The molecular docking data also validated that AB4 could bind to bioactive sites of TLR4. PSC significantly reversed the level of myeloperoxidase and the release of proinflammatory cytokines TNF-α, IL-1β, and IL-6 caused by infection. Therefore, PSC exhibited a potential therapeutic effect on pneumonia via the TLR4/MyD88 pathway [74].

*P. chinensis* extract was found to be highly effective against *Trichomonas vaginalis* at 1–5 h after the exposure to the drugs, and immobilize the parasites completely 2 or 3 days after the exposure [75]. *P. koreana* extract demonstrated a great inhibition against *Toxoplasma gondii* (RH strain) and *N. caninum* (VMDL strain) [76]. *P. chinensis* total saponins have the effects against eggs, miracidia, cercariae of *Schistosoma japonicum *in vitro [77], and also influence the metabolism of parasite by reducing the content of glycogen and decreasing the activity of alkaline phosphatase, acid phosphatase, superoxide dismutase and glutathione reductase [78]. In addition, the total saponins can cause the surface damage of adult worm of *Schistosoma japonicum* [79]. It has been reported that Hederacolchiside A1 (HSA) showed antischistosomal activity against both juvenile and adult *Schistosoma japonicum* with a dose–response relationship. HSA was even more effective than the currently used drugs, praziquantel and artesunate. HSA-mediated antischistosomal activity is partly due to the morphological changes in the tegument system when newly transformed schistosomula are exposed to HSA [80].

## Toxicity

Hepatotoxicity has been of great concern in the clinic and experimental pharmacology studies of *P. chinensis*. 14 of 36 saponins isolated from *P. chinensis* showed considerable cytotoxic activity to human cancer cell lines (A549, SGC-7901) and human hepatic cell line (HL-7702) [81]. Analysis of structure–activity relationships suggested that the free carboxylic group located at C-28 of aglycon and the length and linkage ways of glycolic chain attached to C-3 of aglycon may be essential for the potent cytotoxicity. The acute toxicity test indicated that the powder of PS was considered as practically non-toxic. However, according to the analysis of histopathology, mild lesions in liver and kidney were observed in rats [82]. The UPLC-QTOF-MS was applied to detect multiple time dependent metabolic changes in the serum of rats that treated with PS [83]. 15 biomarkers found in the serum which were closely related to liver injure were changed, including alanine transaminase (ALT), aspartate aminotransferase (AST) and adenosine triphosphate (ATP). Studies also indicated that PS induced ceramide/sphingomyelin (Cer/SM) imbalance, leading to lipid metabolism disorder and cell apoptosis, which gradually damaged the rat’s liver, and ultimately caused chronic liver injury [84]. To overcome the toxicity, vinegar processing was applied to prepare *Pulsatilla* [85]. After vinegar processing, AB4 was increased and accompanied by the decrease of most pentacyclic triterpenoid saponins content. The acidic and heating conditions provided by vinegar allowed PS to undergo three reactions: hydrolysis of oxygen bonds in the ring, reduction of hydroxyl groups on side chain hydrocarbons and acylation of hydroxyl groups on sugar chain. These chemical reactions may change the active groups of pentacyclic triterpene saponins and reduce the disturbance to Cer/SM balance.

These studies suggest that toxicity is one of the main obstacles to the clinical application of PS, and a method like vinegar processing provides a way for subsequent researchers to reduce the toxicity of PS and promote the practical application of PS.

## Conclusion

*P. chinensis* and *P. koreana* are the most studied traditional Chinese medicinal herbs in the genus of *Pulsatilla*. They have been prescribed for thousands of years. To better understand the pharmacological effects and mechanisms for the application of the genus of *Pulsatilla*, plenty of compounds have been extracted for biological activity and mechanistic studies, particularly the saponins, which are considered the major active components in these herbs. There are eight types of saponins based on their aglycons, and 68 kinds of saponins were found in *Pulsatilla* (Fig. 1). Among these compounds, PSA, AB4, PSD, PSE and Raddeanoside R13 have been studied extensively and showed a broad spectrum of bioactivities, such as antitumor, anti-inflammation, antioxidation, immunomodulation, anti-microorganism and organ protection. *Pulsatilla* saponins exert their activities through regulating numerous cellular processes and related signaling pathways as summarized in Fig. 2. Although the molecular mechanisms of those compounds have been verified to a great degree, and some studies revealed that the cytotoxicity of PS may be related to the groups located at C-28 and C-3, the structure–activity relationship (SAR) is still lack of in-depth research and verification. In addition, none of the components, including saponins from these genera have been successfully developed to be a therapeutic agent for clinical application. More in-depth researches on active components, mechanisms of actions, pharmacodynamics, pharmacokinetics, toxicology of the genus of *Pulsatilla* are still highly demanded.

In conclusion, the saponins from the genus of *Pulsatilla* have great medicinal value and application potential. Studies have elucidated the underlying mechanisms for their activities against several diseases at the cellular and animal levels. Although there is lack of preclinical and clinical data to support the application of PS, the potent pharmacological activities of PS with multiple targets suggest that they are promising candidates for therapeutic drug development.

## Data Availability

Not applicable.

## Abbreviations

*P. chinensis*: *Pulsatilla chinensis*
PS: *Pulsatilla* Saponins
AA3: Anemoside A3
PSA: Pulchinenoside A
PSC: Pulchinenoside C
AB4: Anemoside B4
PSD: Pulsatilla saponin D
PSE: Pulsatilloside E
HBA: 23-Hydroxybetulinic acid
HCC: Hepatocellular carcinoma
ATGs: Autophagy-related proteins
LC3: Microtubule-associated protein light chain 3
EMT: Epithelial–mesenchymal transition
HIF-1α: Hypoxia-inducible factor α
VEGF: Vascular endothelial growth factor
PKE: *Pulsatilla koreana* Extract
HUVEC: Human umbilical vein endothelial cells
FASN: Akt/Fatty acid synthase
TNF-α: Tumor necrosis factor α
IL-1β: Interleukin-1β
NF-kB: Nuclear factor kappa B
iNOS: Inducible nitric-oxide synthase
COX-2: Cyclooxygenase-2
MOG: Myelin oligodendrocyte glycoprotein
MDA: Malondialdehyde
HNE: 4-Hydroxy-2-nonenal
ALS: Amyotrophic lateral sclerosis
OVA: Ovalbumin
IFN-γ: Interferon γ
VGCC: L-type voltage-gated Ca^2+^ channel
ROCC: Receptor-operated Ca^2+^ channel
BDNF: Brain-derived neurotrophic factor
NMDA: Non-competitive *N*-methyl-d-aspartate
TLR4: Toll-like receptor 4
MyD88: Myeloid differential protein-88
MD-2: Myeloid differentiation protein-2
ALT: Alanine transaminase
AST: Aspartate aminotransferase
ATP: Adenosine triphosphate
Cer/SM: Ceramide/sphingomyelin
SAR: Structure–activity relationship
